# Refractory depression: mechanisms and evaluation of radically open dialectical behaviour therapy (RO-DBT) [REFRAMED]: protocol for randomised trial

**DOI:** 10.1136/bmjopen-2015-008857

**Published:** 2015-07-17

**Authors:** T R Lynch, B Whalley, R J Hempel, S Byford, P Clarke, S Clarke, D Kingdon, H O'Mahen, I T Russell, J Shearer, M Stanton, M Swales, A Watkins, B Remington

**Affiliations:** 1 Department of Psychology, University of Southampton, Southampton, UK; 2 Department of Psychology, University of Plymouth, Plymouth, UK; 3 Institute of Psychiatry, Psychology & Neuroscience, King's College London, London, UK; 4 Institute for Social and Economic Research, University of Essex, Colchester, UK; 5 University Department of Mental Health, Bournemouth University and Intensive Psychological Therapies Service, Dorset Health Care University NHS Foundation Trust, Poole, UK; 6 Department of Medicine, University of Southampton, Southampton, UK; 7 Mood Disorders Centre, University of Exeter, Exeter, UK; 8 College of Medicine, Swansea University, Swansea, UK; 9 Primary Care and Public Health Sciences, King's College London, London, UK; 10 Psychology Services, Southern Health NHS Foundation Trust, Winchester, UK; 11 School of Psychology, Bangor University, Bangor, UK

**Keywords:** Refractory Depression, Radically Open Dialectical Behaviour Therapy, Study Protocol

## Abstract

**Introduction:**

Only 30–40% of depressed patients treated with medication achieve full remission. Studies that change medication or augment it by psychotherapy achieve only limited benefits, in part because current treatments are not designed for chronic and complex patients. Previous trials have excluded high-risk patients and those with comorbid personality disorder. Radically Open Dialectical Behaviour Therapy (RO-DBT) is a novel, transdiagnostic treatment for disorders of emotional over-control. The REFRAMED trial aims to evaluate the effectiveness and cost-effectiveness of RO-DBT for patients with treatment-resistant depression.

**Methods and analysis:**

REFRAMED is a multicentre randomised controlled trial, comparing 7 months of individual and group RO-DBT treatment with treatment as usual (TAU). Our primary outcome measure is depressive symptoms 12 months after randomisation. We shall estimate the cost-effectiveness of RO-DBT by cost per quality-adjusted life year. Causal analyses will explore the mechanisms by which RO-DBT is effective.

**Ethics and dissemination:**

The National Research Ethics Service (NRES) Committee South Central – Southampton A first granted ethical approval on 20 June 2011, reference number 11/SC/0146.

**Trial registration number:**

ISRCTN85784627.

**Strengths and limitations of this study**

- This study tests a novel and promising psychotherapeutic intervention, specifically designed for patients with chronic depression that is hard to treat.
- Radically Open Dialectical Behaviour Therapy (RO-DBT) is manualised, and is increasingly used for over-controlled patients.
- Our inclusive sample will add general findings to the limited evidence base on interventions for refractory depression.
- Our mechanistic analyses will estimate the role of mediators, including therapeutic alliance and adherence to treatment, by reducing bias inherent in conventional analyses of mediation.
- Our comparator for the effectiveness of RO-DBT is usual clinical practice, including medication and other psychotherapies.

## Introduction

The WHO predicts that by 2020 depression will be the second most frequent cause of disability worldwide.^1^ In the UK, the estimated treatment cost of mood disorders was £25 billion in 2006, some 1.5% of gross domestic product (GDP).^2^ Although there are efficacious treatments for depression, only half of these individuals respond to psychological treatment and only 30–40% of individuals treated with antidepressants achieve full remission.^3^ Treatment resistance is a common outcome for individuals with depression,^3^ and those with chronic depression are least likely to respond to current available treatments.^4^ *Treatment-resistant depression* (TRD) is depression that does not respond to adequate intervention, whereas *chronic depression* is depression lasting more than 2 years. In practice, chronic depression and TRD overlap, with many patients meeting both definitions. The term *refractory depression* (RD) encapsulates both definitions.

Research on intervention for RD is sparse, and most have focused on pharmacological or somatic methods. A recent systematic review of trials of medication for TRD reported many conceptual and methodological problems relating to consensus on defining and assessing RD, or on adequate response to treatment.^3^ Another review identified only four trials of psychotherapeutic treatment for TRD, none of which recruited more than 25 participants.^5^ Most such trials also exclude the most severely disturbed individuals, for example, those with comorbid personality disorder (PD) who are known to respond less favourably to existing treatments for acute depression, notably cognitive behavioral therapy.^4^

Few psychotherapy trials focus on patients with RD because current treatments may not be designed to meet their needs. One exception is Cognitive Behavioral Analysis System of Psychotherapy (CBASP) which was developed specifically to respond to chronic depression.^6^ In one trial, depressed individuals who did not respond to nefazodone, an antidepressant and were randomised to receive CBASP had a better response rate than those who continued to receive nefazodone only.^7^ However, a recent large trial showed when an individualised regimen of antidepressants was augmented by either CBASP or brief supportive psychotherapy (BSP) in partial or non-responders, neither psychotherapy added significant benefit to medication; only 38% derived clinically significant improvement from further medication or psychotherapy.^8^ This study reflects earlier findings from the Sequenced Treatment Alternatives to Relieve Depression study (STAR*D) which found that, among patients who failed to respond to antidepressant medication (ADM), fewer than one-third benefited from adding or switching to, cognitive therapy.^9^ ^10^ Finally, although the Re-ChORD trial showed that a package of group interpersonal psychotherapy, medication and occupational therapy achieved better remission rates than treatment as usual (TAU) at the end of the treatment trial, it lacked data to show that these gains were maintained.^11^

This protocol outlines a randomised controlled trial of Radically Open Dialectical Behaviour Therapy (RO-DBT), a novel psychotherapeutic treatment for RD.^12^ ^13^ RO-DBT arises from neurobiosocial theory^14^ and the observation that current treatments neglect the role of emotional over-control in RD. It proposes that, although the ability to inhibit competing urges, impulses, behaviours and desires is valued by most societies,^15^ too much self-control can be problematic. Excessive self-control has been linked to social isolation, aloof interpersonal functioning, maladaptive perfectionism, disingenuous emotional expression and mental health problems like anorexia nervosa, obsessive-compulsive PD and chronic depression.^16–20^ Of the unipolar depressed patients, 40–60% meet criteria for comorbid PD;^21–23^ over-controlled PDs, notably paranoid, avoidant and obsessive-compulsive, are the most common and least likely to respond to treatment.^4^ ^21^ ^24^ Children exhibiting behavioural over-controlled traits like shyness, timidity, restrained emotional expression and risk aversion are more likely to develop internalising disorders and become socially isolated adults.^16^ ^17^ ^25^ ^26^ Adults with chronic depression are characterised by over-controlled traits, including greater self-criticism, impaired autonomy, rigid internalised expectations, excessive control of spontaneous emotion and inordinate fears of making mistakes.^23^

The neurobiosocial theory underlying RO-DBT posits that individuals presenting with problems of over-control are biologically predisposed to exhibit heightened threat sensitivity, diminished reward sensitivity, strong tendencies towards constraint and preoccupation with details.^14^ Heightened biotemperamental threat sensitivity predisposes a person to prioritise the *potential for harm* over the *potential for reward* when entering new or unfamiliar situations, thereby activating sympathetic nervous system (SNS) defensive-arousal and fight–flight responses. These perceptual biases are strengthened by family or environmental histories reinforcing avoidance of risk and masking of emotions. Heightened SNS arousal also triggers withdrawal of social safety engagement responses mediated by the parasympathetic nervous system ventral vagal complex (PNS-VVC).^27^ Unfortunately, when the PNS-VVC is withdrawn and SNS-mediated flight–fight responses dominate, neuroregulatory responses linked with prosocial engagement are impaired, facial expressions become frozen and the ability to express oneself flexibly is lost.^27^ ^28^ Inhibited or incongruent expressions of emotion are perceived as inauthentic or untrustworthy by others and thus increases social ostracism.^29^ ^30^ Since these responses may create or maintain depressive symptoms, RO-DBT uniquely emphasises both *communicative* and *facilitative* functions of emotional expressions in the formation of close social bonds and empathic behaviours via micromimicry and the mirror neuron system.^12^ ^13^ ^31^ Therapy includes training patients to relax rigid inhibitory self-control efforts, to activate the VVC-mediated social-safety system, to use non-verbal social-signalling skills linked to the mirror neuron system and the establishment of trust to allow vulnerable self-disclosure, to practice self-enquiry to learn from new experiences and critical feedback, and to reduce maladaptive social comparisons related to envy and bitterness.

Earlier versions of RO-DBT showed promise in two trials of patients with refractory depression and comorbid PD.^32^ ^33^ The manualised version of RO-DBT^12^ has also been effective in two open trials of adults with anorexia nervosa.^34^ ^35^

### Objectives

Our primary aim is to estimate the effectiveness of RO-DBT in treating RD, compared with TAU. We shall also estimate the cost-effectiveness of RO-DBT relative to TAU alone. We also plan to study moderators of treatment effects and mechanisms of therapeutic change. As RO-DBT emphasises the therapist's role as a model of interpersonal skills, we predict that the development and maintenance of a strong therapeutic alliance will mediate reductions in depressive symptoms. Similarly, since the treatment is challenging, we regard adherence as an important potential mediator.

## Methods

### Design and setting

REFRAMED is a multicentre, randomised controlled trial conducted at three National Health Service (NHS) sites in the UK—Dorset, Hampshire and North Wales. These sites routinely offer outpatient psychological treatment. All trial participants receive TAU, but those allocated at random to the experimental arm also receive 7 months of RO-DBT. We also allocate participants at random between non-therapeutic alternatives designed to facilitate mediational analyses. We measure clinical outcomes at four time points—baseline, and 7, 12 and 18 months after randomisation, using assessors blind to participants’ allocated treatment.

### Participants

#### Inclusion criteria

Participants must be 18 years or older, and have a Hamilton-Depression Rating Scale (HAM-D)^36^ score of at least 15, a current diagnosis of major depressive disorder in the Structured Clinical Interview for DSM-IV Axis I (SCID-I),^37^ and *either* TRD defined as two or more previous episodes of depression *or* chronic depression. In their current episode, furthermore, participants must have taken an adequate dose of ADM for at least 6 weeks without relief.

#### Exclusion criteria

We exclude those who meet criteria for dramatic–erratic PD (Cluster B), bipolar depression or psychosis, or have a primary diagnosis of substance dependence or substance abuse disorder. Patients must have an IQ of more than 70 and speak English well enough to participate in the research, including treatment.

### Procedures

**Figure 1:**
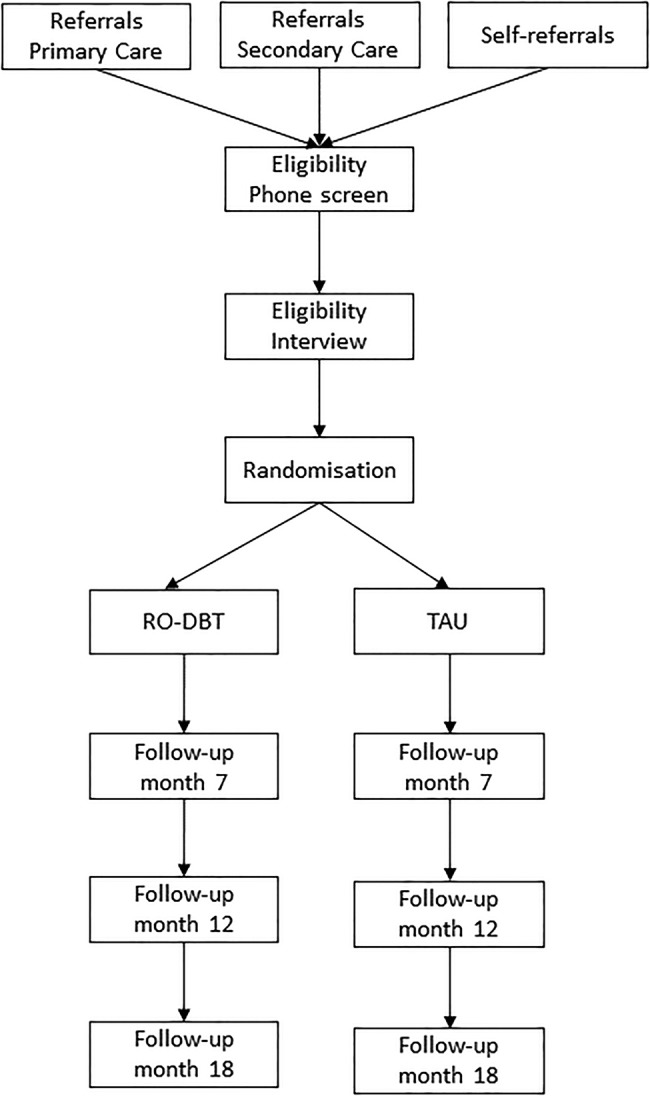
CONSORT diagram of REFRAMED study participant recruitment, assessment and follow-up timeline.

Please see figure 1 for a CONSORT diagram of the REFRAMED study participant recruitment, assessment and follow-up timeline.

### Recruitment

We recruit participants through mental health clinicians, general practitioners (GPs) and advertisements in clinics and community. Clinical study officers within local NHS organisations handle enquiries and screen potential participants for inclusion criteria and the absence of exclusion criteria by telephone, using a standardised checklist. If eligible and willing, participants attend a second assessment interview.

### Eligibility

Our team of trained assessors establish whether these potential participants are indeed eligible. They also administer the baseline assessment of the outcome measures set out in table 1. After the interview, participants receive further questionnaires for home completion. Our assessment lead (HO) monitors interassessor reliability, in particular whether κ’s for SCID and HAM-D are consistently at least 0.70.

**Table 1:** Assessment schedule and overview of measures

	Measurement occasion													
	*Baseline*	*Treatment*						*Follow-up*						
Month	0	1	2	3	4	5	6	7	8	9	10	11	12	18
*Primary outcomes*														
Depression (HAM-D, LIFE-RIFT)	•							•					•	•
Health-related quality of life (EQ-5D-3 L)	•			•				•					•	•
Health services use/costs (AD-SUS)	•							•					•	•
*Secondary outcomes*														
Suicide (MSSI, SBQ)	•							•					•	•
Depression and affect (PHQ-9, PANAS)	•	•	•	•	•	•	•	•	•	•	•	•	•	•
*Mechanisms and mediators*														
Psychosocial function(WBSI, SSQ3, IIP-PD-25, DBT-WCCL)	•			•				•					•	•
Emotional approach and expectancy (EAC, CEQ)	•	•	•	•	•	•	•	•	•	•	•	•	•	•
Alliance and delivery of treatment (CALPAS, CSQ-8, sessions attended)	•	•	•	•	•	•	•		•					
*Moderators of RO-DBT effectiveness*														
Personality and personality disorder (SCID-I and II, NEO-FFI-C, applied conscientiousness task, SVS)	•													
Temperament and emotional control (UPPS, PNS, Ego-undercontrol, Ego-resiliency, BIDR-SF, FMPS)	•													
Childhood experience and invalidation (ICES, MOPS)	•													

AAQ-II, Acceptance and Action Questionnaire-II;^38^ AD-SUS, Adult Service Use Schedule;^39^ AEQ, Ambivalence over Emotional expression Questionnaire;^40^ BIDR-SF, Balanced Inventory of Desirable Responding-Short Form (Hart CM, Ritchie T, Hepper EG, *et al* The Balanced Inventory of Desirable Responding Short Form: Reliability, validity and factor structure. submitted manuscript 2012);CALPAS, California Psychotherapy Alliance Scales;^41^ CEQ, Credibility/Expectancy Questionnaire;^42^ CSQ-8, Client Satisfaction Questionnaire–8;^43^ DBT-WCCL, Dialectical Behavior Therapy Ways of Coping Checklist;^44^ EAC, Emotional Approach Coping scale;^45^ EQ-5D-3L, European Quality of Life Questionnaire—5 Dimensions;^46^ FMPS, Frost Multidimensional Perfectionism Scale;^47^ HAM-D, Hamilton Rating Scale for Depression;^36^ ICES, Invalidating Childhood Experiences Scale;^48^ IIP-PD-25, Inventory of Interpersonal Problems-Personality Disorders-25 items;^49^ LIFE-RIFT, the Longitudinal Interval Follow-up Evaluation—Range of Impaired Functioning Tool;^50^ MOPS, Measure of Parenting Style;^51^ MSSI, Modified Scale for Suicide Ideation;^52^ NEO-FFI, NEO Five Factor Inventory;^53^ PANAS, Positive and Negative Affect Scale;^54^ PHQ-9, Patient Health Questionnaire-9;^55^ PNS, Personal Need for Structure;^56^ SBQ, Suicidal Behaviors Questionnaire;^57^ SCID-I and SCID-II, Structured Clinical Interviews for DSM-IV;^37^ ^58^ SCS, Self-Compassion Scale;^59^ SSQ3, Social Support Questionnaire;^60^ SVS, Schwartz Values Scale;^61^ UPPS, Urgency Premeditation Perseverance Sensation Seeking scale;^62^ WBSI, White Bear Suppression Inventory.^63^

### Randomisation

Once we receive all baseline questionnaires, we allocate each participant between treatments through an adaptive randomisation algorithm administered by Swansea Trials Unit. This maintains the balance across groups of three stratifying variables chosen as potential outcome moderators, but stochastically rather than deterministically to minimise risk of subversion:^64^

- Early onset of depression, that is, before 21 years of age
- Depression severe at baseline (HAM-D above 25)
- Personality disorder (meets SCID-II criteria for cluster A or cluster C)

We also use adaptive randomisation to allocate patients between therapists so as to use as many feasible treatment slots at each centre. The allocation sequence was determined dynamically using a database independently administered at the study's Clinical Trials Unit; the database incorporated a study-specific version of a published dynamic randomisation allocation method,^65^ and the resulting allocations were then sent by email to the Trial Manager for further dissemination to study therapists.

#### Mediation and instrumental variables

To understand the mechanisms by which RO-DBT improves outcomes, we shall conduct mediation analyses to estimate the effects of postrandomisation measures on the pathway from randomisation to outcome. Conventional mediation analyses may be biased by unobserved confounding, that is confounding by unobserved variables related to both the mediators and the outcomes.^66^ ^67^ Confounding arises because, following randomisation to treatment arm, patients choose the manner in which they adhere to treatment and the level of adherence; if one patient adheres in a certain way and coincidentally suffers worse depression, confounding ensues. To address unobserved confounding, we shall use ‘instrumental variables’ (IVs) which:

1. Predict the likelihood that a patient will adhere to treatment;
2. Are unrelated to the study outcome; and
3. Are unrelated to the unobserved confounding variables.

If we can find one or more IVs, we can use methods like two-stage least squares to estimate the unconfounded effect of the mediator. The biggest challenge is to find IVs. Some occur naturally; for example, genes can serve as IVs: one variant of the ALDH2 gene makes it unpleasant for individuals to drink alcohol, facilitating unbiased estimates of the effect of alcohol on health from observational data.^68^ Alternatively, researchers make additional random allocations between options that are not therapeutic in their own right, but may influence mediators;^69^ for example, enhancing the therapeutic environment may enhance alliance (the mediator), but have no direct effect on symptoms (the outcome).

REFRAMED has adopted both approaches to facilitate bias-corrected estimates of the effects of two hypothesised mediators: *strength of therapeutic alliance* and *adherence to treatment.* We randomly allocate all RO-DBT participants to one of eight combinations of contextual non-therapeutic features within a 2×2×2 factorial design:

- They receive individual RO-DBT in either a *standard therapy room* or an *enhanced therapy room,* which potentially improves both strength of therapeutic alliance and attendance at treatment;
- After every session they either *have* or *do not have opportunity to provide written feedback* to *their therapist,* which improves strength of therapeutic alliance;^70^ and
- They receive compensation potentially for questionnaire completion either *personally* (via their therapists) or *impersonally* (via mail), again potentially increasing attendance at treatment.

While we recognise that these ploys may have limited influence on our adopted mediators,^71^ we include them both for their potential to reduce bias in our estimates, and to encourage the wider adoption and discussion of these methods in psychotherapy research.

### Intervention phase (29 weeks)

#### Treatment as usual

This control intervention permits participants to receive any psychotherapy or prescribed (ADM) with the exception of standard DBT. We monitor type and amount of psychotherapy, and type of ADM and adherence to it. Reporting these data will better describe this generic intervention. In particular, we do not restrict access to appropriate mental healthcare during follow-up.

#### Radically open-dialectical behavior therapy

This experimental intervention comprises 29 weekly individual therapy sessions lasting 50–60 min, and 27 weekly skills training classes lasting 2.5 h, including a 15 min break (table 2). RO-DBT therapists meet weekly for 1.5–2.5 h, and by telephone when needed.^12^ ^13^ While RO-DBT participants receive ADM as prescribed, we strongly discourage them from seeking additional psychotherapy during RO-DBT. The RO-DBT treatment developer and study chief investigator (TRL) does not contribute to the treatment.

**Table 2:** RO-DBT treatment goals and target hierarchy for over-controlled disorders

Primary targets and goals DECREASE severe behavioural over-control, emotional loneliness and aloofness/distance INCREASE openness, flexibility, prosocial signalling and vulnerable expression of emotion	
**Treatment target hierarchy for over-control**	
1. Life-threatening behaviours	**Suicidal ideation and non-suicidal self-injury** *Over-controlled self-injurious behaviour tends to be planned in advance, occurs in private and rarely requires immediate medical attention*
2. Therapeutic alliance ruptures	**Patient feels misunderstood or perceives therapy as not relevant to their problems** *Signals of non-engagement by over-controlled patients tend to be understated, for example saying “hmm”, “maybe” or “I guess so” when disagreeing, or by avoiding eye contact or changing the topic when feeling misunderstood*
3. Over-controlled Behavioural themes	**Constrained expressions of emotion** *Over-controlled patients tend to display inhibited, flattened or insincere facial expressions (eg, smiling when distressed, showing concern when not feeling it), have a monotonic voice tone and tight and non-expansive gestures or body movements. They will work hard to avoid public displays of emotion* **Overly cautious and hyper-vigilant** *Over-controlled patients are not necessarily avoidant but guarded, wary and suspicious. They exhibit superior detailed-focused processing* **Rigid and rule-governed behaviours** *The actions of over-controlled patients tend to be non-mood dependent and instead follow certain self-imposed rules. They are motivated by social obligation and exhibit high moral certitude; they often make self-sacrifices to care for others or to do the ‘right’ thing. They tend to be hyper-perfectionistic and have compulsive needs for order and structure* **Aloof and distant relationships** *Over-controlled patients do not necessarily lack contact but lack social connectedness with others. They are slow to warm-up and will walk away or abandon a relationship when in conflict. They are likely to feel like an outsider, different or detached from others* **Envy, Resentment, Bitterness and Revenge** *Over-controlled patients tend to be performance-focused, engage in social comparisons, are secretly competitive, tend to hold grudges and may have secret pride in superior capacities for self-control. They may take pleasure in a rival failing or feel unappreciated for personal self-sacrifices or efforts on their part to meet or exceed expectations*

Targets higher in the treatment hierarchy take priority over lower ones. Thus, life-threatening behaviors and therapeutic alliance-ruptures take precedence over behavioral themes when these are present. Therapists use the behavioral themes to facilitate treatment planning.

#### Therapist training and supervision

All therapists had to complete a two-part, 10-day RO-DBT intensive training course over 6 months and additional training or supervision when needed to achieve adherence to the treatment manual. We videotape all treatment sessions and shall sample 10% of both therapy sessions and skills classes at random, stratified by therapist and participant, for adherence rating.

### Discontinuations

If participants are unable or unwilling to attend RO-DBT sessions, we consider them as treatment drop-outs but encourage them to provide outcome data. If they withdraw from data collection, we permit them to continue with RO-DBT. If they withdraw consent to participate in the study, however, we do not seek further data.

### Outcome measures

Table 1 summarises the schedule of outcome measures.

### Primary outcome

The primary outcome is depressive symptoms, measured on the 17-item Hamilton Rating Scale for Depression (HAM-D)^36^ at four points (baseline, and 7, 12 and 18 months after randomisation) by assessors who report their ‘blindness’ after each assessment. We replace them with unblinded assessors for subsequent assessments.

### Secondary outcomes

Secondary outcomes at the same four points include remission status, and suicidal ideation and behaviour. We define remission as the combination of a HAM-D score of less than 8 and minimal functional impairment on the Longitudinal Interval Follow-up Evaluation—Range of Impaired Functioning Tool (LIFE-RIFT);^50^ we measure suicidal thoughts and behaviours on the Modified Scale for Suicidal Ideation^52^ and the Suicidal Behaviors Questionnaire.^57^

### Health economics data

We collect health economic data alongside our primary outcomes. For health costs we use the Adult Service Use Schedule (AD-SUS); and for health utility the European Quality of Life Questionnaire (EQ-5D-3 L).^46^ The AD-SUS measures medication and service use, and lost productivity due to illness, in mental health populations, including patients with depression.^39^ We also ask therapists about unscheduled time in treating RO-DBT patients, notably calls out of hours.

### Mediators of outcome

We collect data on potential outcome mediators throughout the treatment and follow-up using questionnaires to measure interpersonal, emotional, and psychosocial functioning, strength of therapeutic alliance, use of skills taught in treatment and expectancy (table 1).

Between these questionnaires, we collect data on mood, emotional coping and the credibility of and expectancy attached to the intervention, once a month for the first 12 months and again at 18 months. We also use an automated telephone system to measure mood (PANAS Short-Form),^72^ coping skills (6 items from the DBT-WCCL)^44^ and self-compassion (Self-Compassion Scale)^59^ once a week over the first 6 months that participants are in the trial.

### Moderating variables

At baseline we collect data on potential moderating variables, including basic demographics and Axis I and Axis II disorders, childhood experiences and invalidation, and personality and markers of personality disorder (table 1). To control for social desirability, we ask participants to complete the Balanced Inventory of Desirable Responding-Short Form. (Hart CM, Ritchie T, Hepper EG, *et al*. The Balanced Inventory of Desirable Responding Short Form: Reliability, validity, and factor structure. submitted manuscript 2012).

### Characteristics of therapists

To investigate whether therapist characteristics, and their relationship with patient characteristics influence outcome, we collect measures of perfectionism (Frost Multidimensional Perfectionism Scale),^47^ personal values (Schwartz Values Scale)^61^ and attachment experiences (Measure of Parenting Style).^51^ To provide an instrumental variable for strength of therapeutic alliance, we measured therapists’ capacity to generate strong alliances before the trial by asking several patients from the caseload of each therapist to complete the California Therapeutic Alliance Scale.^41^

### Sample size estimation

Based on an average effect sizes close to one from two RO-DBT pilot trials for RD^32^ ^33^ and one pilot trial of DBT for TRD,^73^ we judged it feasible and desirable to power REFRAMED to detect a standardised difference of 0.4 between-groups (RO-DBT and TAU)—likely to be considered clinically relevant by the UK National Institute of Health and Care Excellence (NICE).

In the absence of intraclass correlation, a sample of 200 analysable participants from the three centres would yield 80% power when using a significance level of 5% to detect a mean difference of two points on the HAM-D, equivalent to a standardised difference of 0.4. Since we expect to collect analysable data from 83% of participants, we increased our initial target to 240. To increase the power of our mechanistic analyses, we planned to randomise 144 of these to RO-DBT. As participants receiving RO-DBT are clustered by therapist, we increased the target RO-DBT sample size from 144 to 180 to allow for an intracluster correlation coefficient of 0.025 for HAM-D scores, and an average cluster size of 11. Thus we aim to randomise 276 patients—180 to RO-DBT and 96 to TAU (a final allocation ratio of 15:8).

### Statistical analyses

For our primary measure of depression (HAM-D), we shall analyse by treatment allocated and use a mixed-effects model to estimate the effectiveness of RO-DBT. This will include random effects to account for clustering of outcomes by therapist, and fixed effects to account for treatment allocation, differences between sites and their interaction. In particular, this model does not assume that all therapists are equally effective.^74^ Covariates will include baseline HAM-D score, diagnosis of PD at baseline and age at first onset of depression. Repeated HAM-D measurements enable us to compare RO-DBT and TAU at 7, 12 and 18 months after randomisation. Secondary analyses will test for interaction between treatment allocation and covariates, and estimate the marginal effect of RO-DBT within strata. If data are missing at random (MAR), these models will be unbiased and efficient. We shall, therefore, analyse missing data thoroughly and qualify our conclusions accordingly.

### Mechanisms, instrumental variables and causal analyses

Initially our causal analysis will consider each potential mediator in turn, estimating both the direct effect of treatment via exposure, and the indirect effect via the mediator. Following Baron and Kenny,^75^ we shall compare the resulting estimates with those from a naïve analysis that assumes no unobserved confounding, and state the assumptions under which the causal estimates are unbiased and consistent. Finally we shall extend these analyses to a joint model of all mediators and IVs. To explore patterns of temporal ordering from skill acquisition, through development of the therapeutic alliance, to changes in depressive symptoms, we shall use multivariate growth curve models including autoregressive and lagged terms.

### Economic evaluation

We shall undertake cost-utility analysis to estimate the cost effectiveness of RO-DBT relative to TAU alone in terms of cost per Quality-Adjusted Life Year (QALY) derived from the EQ-5D-3L. We shall use cost-effectiveness analysis of depressive symptoms from the HAM-D as sensitivity analysis. Our primary perspective will be that of the NHS and personal social services, as preferred by the National Institute for Health and Care Excellence (NICE).^76^ Again we shall consider societal losses due to lost productivity, including both absence from work and reduced productivity during work, in the sensitivity analysis. We shall estimate cost-effectiveness through both approaches used to estimate clinical effectiveness: conventional analysis by treatment allocated, and IV analysis based on treatment received, that is adherence. We shall use non-parametric bootstrapping to generate the joint distribution of mean incremental costs and effects of RO-DBT relative to TAU and thus, explore the probability that one treatment is better, given NICE's ‘willingness-to-pay’ threshold of £20 000 to £30 000 per QALY. We shall summarise uncertainty around these estimates by cost-effectiveness acceptability curves.^77^

### Data collection and management

#### Paper data

To ensure accuracy, completeness and reliability, trained assessors collect outcome data during interviews in person or by phone by following standard operating procedures for data collection and transfer to the trial office in Southampton. Each month we post additional paper questionnaires direct to participants with a free return envelope, and send text or email reminders after 10 days.

#### Electronic data capture system

We enter all paper data twice onto Signalbox (http://www.thesignalbox.net), a validated electronic data capture system. This system also collects the data from automated phone calls and therapists’ treatment notes online, notably by issuing weekly electronic reminders.

#### Study monitoring

The Trial Steering Committee (TSC) and Data Monitoring Committee (DMC) monitor the trial to ensure we comply with the rigorous standards defined in the Medical Research Council's (MRC) Guidelines for Good Clinical Practice. The TSC meets twice a year throughout the trial. The DMC met twice in the first year and annually since then. Both the TSC and DMEC are independent from the Funder and Sponsor.

### Reporting of adverse events and study termination

Site principal investigators (PIs) monitor and assess serious adverse events (SAEs) and report them to the chief investigator immediately, and to the DMC regularly, or immediately when the SAE is a Suspected Unexpected Serious Adverse Reaction (SUSAR). Site PIs discuss SAEs with the relevant participants and their GPs with their permission. The trial will terminate if the TSC, on the recommendation of the DMC or the chief investigator, judges it necessary for the welfare of trial participants or for the scientific validity of the trial.

### Ethical considerations and dissemination

We ask trial participants to give consent on three occasions:

1. Oral consent to the phone screen;
2. Signed consent for the baseline assessment, stating that they have read and understood the information sheet and giving permission for the interview to be audio recorded;
3. Eligible participants sign the reverse of their consent forms, stating that they understand the patient information sheet and are willing to participate in the trial.

Personal information will kept on secure databases (one for each site), only accessible to members of staff who need these details for making assessment appointments and sending letters. These are the only files that link personal information with the participant identification numbers.

We conduct REFRAMED in accordance with the Declaration of Helsinki. We have received approval from the Hampshire Research Ethics Committee (National Research Ethics Service (NRES) reference number 11/SC/0146) and the Ethics & Research Governance Department of the University of Southampton, the Sponsor. REFRAMED has insurance cover under the Sponsor's Professional Indemnity and Clinical Trials policy. All investigators comply with REFRAMED's policy on conflicts of interest, and will report conflicts of interest in all resulting publications.

We shall implement and report REFRAMED in accordance with all relevant CONSORT guidance. We plan to publish the findings in high-impact, peer-reviewed journals and disseminate them to service providers and users across the UK and beyond via public forums and websites. In accordance with Research Council guidelines, we shall store anonymised individual patient data from REFRAMED in a repository accessible by other researchers following publication.

## Discussion

REFRAMED is a randomised controlled trial of a promising treatment for refractory depression with implications for other diagnoses. It will contribute to the clinical literature in three ways:

1. Our broad selection criteria include patients routinely excluded from depression trials, namely those with comorbid personality disorder, suicidal behaviour, non-suicidal self-injury, prior psychotherapy, frequent relapse or older age. Hence the evidence base is weak for these patients in most need of effective treatment. Our inclusion criteria may mean that this trial will have a higher rate of reported suicide attempts than trials that exclude patients with PDs, comorbid disorders and suicidal ideation. However, our use of open criteria reflects the growing recognition that symptoms of many mental health conditions may share a common aetiology, and that to exclude patients with comorbidities significantly undermines the generalisability and utility of clinical trials.
2. RO-DBT offers a major change in approach to the treatment of refractory depression. Most important is a novel focus on behavioural over-control as a core aetiological factor. The treatment proposes a new mechanism of therapeutic change by linking *neuoregulatory theory* and the *communicative functions of emotional expression* to the *formation of close social bonds.* This translates into novel skills focused on social signalling and changing psychophysiological arousal—a key component differentiating RO-DBT from other treatments.
3. REFRAMED is the first mental health trial to use instrumental variables to analyse causes of mediation. In particular, we have randomised RO-DBT participants between eight combinations of three contextual features that are not intended to be therapeutic in their own right, but may affect mediating variables, including strength of therapeutic alliance and adherence to the treatment. This approach will yield estimates of the effects of mediating variables that are less biased by confounding than conventional estimates. Thus it has the potential to resolve a longstanding criticism of the literature that links strength of therapeutic alliance with outcome—that patients most likely to recover are those most able to form strong alliances.

### Design implications and limitations

REFRAMED does not use an active comparator. Instead, patients in the control arm may access any type of treatment available through the NHS or private healthcare. NICE guidelines suggest combined approaches like ADM and CBT for moderate to severe depression or depression not responding to first-line treatments.^76^ However, ADM alone is more readily available and often preferred for those with unresponsive depression.^8^ Moreover, there is little evidence whether augmenting ADM with psychotherapy is more or less effective than switching between ADMs. Indeed NICE recommendations stem from one multisite study designed to compare switching and augmentation following poor response to acute ADM treatment, but whose findings do not justify firm recommendations.

### Current progress

REFRAMED has recruited participants from March 2012 until May 2015, and will complete data collection by June 2016. All trial therapists have achieved good adherence to the manual. During the pilot phase, the leading site in Dorset achieved three other critical targets by: recruiting 20 participants over 6 months; ensuring that 70% of these responded in full to the primary outcome; and showing that they were satisfied with treatment according to the Client Satisfaction Questionnaire–8.^43^ These achievements enabled the TSC to approve the trial in full.

## References

[1] Murray CJ, Lopez AD Alternative projections of mortality and disability by cause 1990–2020: Global Burden of Disease Study. Lancet 1997;349:1498–504. 10.1016/S0140-6736(96)07492-2 9167458

[2] Layard R, Clark D, Knapp M Implementing the NICE guidelines for depression and anxiety. A cost-benefit analysis. London School of Economics website, 2006.

[3] Berlim MT, Turecki G What is the meaning of treatment resistant/refractory major depression (TRD)? A systematic review of current randomized trials. Eur Neuropsychopharmacol 2007;17:696–707. 10.1016/j.euroneuro.2007.03.009 17521891

[4] Fournier JC, DeRubeis RJ, Shelton RC Prediction of response to medication and cognitive therapy in the treatment of moderate to severe depression. J Consult Clin Psychol 2009;77:775–87. 10.1037/a0015401 19634969 PMC2810269

[5] McPherson S, Cairns P, Carlyle J The effectiveness of psychological treatments for treatment-resistant depression: a systematic review. Acta Psychiatr Scand 2005;111:331–40. 10.1111/j.1600-0447.2004.00498.x 15819726

[6] McCullough JP Jr Treatment for chronic depression: cognitive behavioral analysis system of psychotherapy (CBASP). Educational Publishing Foundation, 2003. 10.1002/jclp.10176 12858425

[7] Keller MB, McCullough JP, Klein DN A comparison of nefazodone, the cognitive behavioral-analysis system of psychotherapy, and their combination for the treatment of chronic depression. N Engl J Med 2000;342:1462–70. 10.1056/NEJM200005183422001 10816183

[8] Kocsis JH, Gelenberg AJ, Rothbaum BO Cognitive behavioral analysis system of psychotherapy and brief supportive psychotherapy for augmentation of antidepressant nonresponse in chronic depression: the REVAMP trial. Arch Gen Psychiatry 2009;66:1178–88. 10.1001/archgenpsychiatry.2009.144 19884606 PMC3512199

[9] Rush AJ, Trivedi MH, Wisniewski SR Acute and longer-term outcomes in depressed outpatients requiring one or several treatment steps: a STAR*D report. Am J Psychiatry 2006;163:1905–17. 10.1176/appi.ajp.163.11.1905 17074942

[10] Thase ME, Friedman ES, Biggs MM Cognitive therapy versus medication in augmentation and switch strategies as second-step treatments: A STAR*D report. Am J Psychiatry 2007;164:739–52. 10.1176/ajp.2007.164.5.739 17475733

[11] Murray G, Michalak EE, Axler A Relief of Chronic or Resistant Depression (Re-ChORD): a pragmatic, randomized, open-treatment trial of an integrative program intervention for chronic depression. J Affect Disord 2010;123:243–8. 10.1016/j.jad.2009.10.015 19896200

[12] Lynch TR Radically open dialectical behavior therapy for disorders of overcontrol. New York: Guilford Press, in press.

[13] Lynch TR, Hempel RJ, Dunkley C Remembering our tribal nature: radically open-dialectical behavior therapy for disorders of overcontrol. Am J Psychother in press. 10.1176/appi.psychotherapy.2015.69.2.141 26160620

[14] Lynch TR, Hempel RJ, Clark LA Radical openness: facilitating self-inquiry in over-controlled personality disorders. In: Livesley J, Dimaggio G, Clarkin J, eds. Integrated modular treatment for personality disorder. New York: Guilford Publications, Inc., in press.

[15] Moffitt TE, Arseneault L, Belsky D A gradient of childhood self-control predicts health, wealth, and public safety. Proc Natl Acad Sci USA 2011;108:2693–8. 10.1073/pnas.1010076108 21262822 PMC3041102

[16] Asendorpf JB, Denissen JJA, van Aken MAG Inhibited and aggressive preschool children at 23 years of age: personality and social transitions into adulthood. Dev Psychol 2008;44:997–1011. 10.1037/0012-1649.44.4.997 18605830

[17] Chapman BP, Goldberg LR Replicability and 40-year predictive power of childhood ARC types. J Pers Soc Psychol 2011;101:593–606. 10.1037/a0024289 21744975 PMC3160513

[18] Eisenberg N, Fabes RA, Guthrie IK Dispositional emotionality and regulation: their role in predicting quality of social functioning. J Pers Soc Psychol 2000;78:136–57. 10.1037/0022-3514.78.1.136 10653511

[19] Anderluh M, Tchanturia K, Rabe-Hesketh S Lifetime course of eating disorders: design and validity testing of a new strategy to define the eating disorders phenotype. Psychol Med 2009;39:105–14. 10.1017/S0033291708003292 18377676

[20] Chapman AL, Lynch TR, Rosenthal MZ Risk aversion among depressed older adults with obsessive compulsive personality disorder. Cognitive Therapy and Research 2007;31:161–74. 10.1007/s10608-006-9114-x

[21] Fava M, Farabaugh AH, Sickinger AH Personality disorders and depression. Psychological medicine: a journal of research in psychiatry and the allied sciences 2002;32:1049–57. 10.1017/S0033291702005780 12214786

[22] Klein DN, Riso LP, Donaldson SK Family study of early-onset dysthymia: mood and personality disorders in relatives of outpatients with dysthymia and episodic major depression and normal controls. Arch Gen Psychiatry 1995;52:487–96. 10.1001/archpsyc.1995.03950180073010 7771919

[23] Riso LP, Blandino JA, Penna S Cognitive aspects of chronic depression. J Abnorm Psychol 2003;112:72–80. 10.1037/0021-843X.112.1.72 12653415

[24] Candrian M, Schwartz F, Farabaugh A Personality disorders and perceived stress in major depressive disorder. Psychiatry Res 2008;160:184–91. 10.1016/j.psychres.2007.06.014 18573540 PMC2553350

[25] Caspi A The child is father of the man: personality continuities from childhood to adulthood. J Pers Soc Psychol 2000;78: 158–72. 10.1037/0022-3514.78.1.158 10653512

[26] Markon KE, Krueger RF, Watson D Delineating the structure of normal and abnormal personality: an integrative hierarchical approach. J Pers Soc Psychol 2005;88:139–57. 10.1037/0022-3514.88.1.139 15631580 PMC2242353

[27] Porges SW The polyvagal theory: phylogenetic substrates of a social nervous system. Int J Psychophysiol 2001;42:123–46. 10.1016/S0167-8760(01)00162-3 11587772

[28] Porges SW The Polyvagal theory: phylogenetic contributions to social behavior. Physiol Behav 2003;79:503–13. 10.1016/S0031-9384(03)00156-2 12954445

[29] Butler EA, Egloff B, Wlhelm FH The social consequences of expressive suppression. Emotion 2003;3:48–67. 10.1037/1528-3542.3.1.48 12899316

[30] Mauss IB, Shallcross AJ, Troy AS Don't hide your happiness! Positive emotion dissociation, social connectedness, and psychological functioning. J Pers Soc Psychol 2011;100:738–48. 10.1037/a0022410 21280962 PMC3265161

[31] Schneider KG, Hempel RJ, Lynch TR That “poker face” just might lose you the game! The impact of expressive suppression and mimicry on sensitivity to facial expressions of emotion. Emotion 2013;13:852–66. 10.1037/a0032847 23795586

[32] Lynch TR, Cheavens JS, Cukrowicz KC Treatment of older adults with co-morbid personality disorder and depression: a dialectical behavior therapy approach. Int J Geriatr Psychiatry 2007;22:131–43. 10.1002/gps.1703 17096462

[33] Lynch TR, Morse JQ, Mendelson T Dialectical behavior therapy for depressed older adults: a randomized pilot study. Am J Geriatr Psychiatry 2003;11:33–45. 10.1097/00019442-200301000-00006 12527538

[34] Chen EY, Segal K, Weissman J Adapting dialectical behavior therapy for outpatient adult anorexia nervosa-a pilot study. Int J Eat Disord 2015;48:123–32. 10.1002/eat.22360 25346237 PMC5670741

[35] Lynch TR, Gray KL, Hempel RJ Radically open-dialectical behavior therapy for adult anorexia nervosa: feasibility and outcomes from an inpatient program. BMC Psychiatry 2013;13:293–00. 10.1186/1471-244X-13-293 24199611 PMC3875355

[36] Williams JBW, Kobak KA, Bech P The GRID-HAMD: standardization of the Hamilton Depression Rating Scale. Int Clin Psychopharmacol 2008;23:120–9. 10.1097/YIC.0b013e3282f948f5 18408526

[37] First MB, Spitzer RL, Gibbon M Structured clinical interview for DSM-IV-TR axis I disorders, research version, patient edition. (SCID-I/P). New York: Biometrics Research, New York State Psychiatric Institute, 2002.

[38] Bond FW, Hayes SC, Baer RA Preliminary Psychometric Properties of the Acceptance and Action Questionnaire–II: a revised measure of psychological inflexibility and experiential avoidance. Behav Ther 2011;42:676–88. 10.1016/j.beth.2011.03.007 22035996

[39] Kuyken W, Byford S, Taylor RS Mindfulness-based cognitive therapy to prevent relapse in recurrent depression. J Consult Clin Psychol 2008;76:966–78. 10.1037/a0013786 19045965

[40] King LA, Emmons RA Conflict over emotional expression: psychological and physical correlates. J Pers Soc Psychol 1990;58:864–77. 10.1037/0022-3514.58.5.864 2348373

[41] Marmar CR, Weiss DS, Gaston L Toward the validation of the California Therapeutic Alliance Rating System. Psychological assessment 1989;1:46–52. 10.1037/1040-3590.1.1.46

[42] Devilly GJ, Borkovec TD Psychometric properties of the credibility/expectancy questionnaire. J Behav Ther Exp Psychiatry 2000;31:73–86. 10.1016/S0005-7916(00)00012-4 11132119

[43] Attkisson CC, Zwick R The client satisfaction questionnaire: psychometric properties and correlations with service utilization and psychotherapy outcome. Eval Program Plann 1982;5:233–7. 10.1016/0149-7189(82)90074-X 10259963

[44] Neacsiu AD, Rizvi SL, Vitaliano PP The Dialectical Behavior Therapy ways of coping checklist: development and psychometric properties. J Clin Psychol 2010;66:563–82. 20455249 10.1002/jclp.20685

[45] Stanton AL, Kirk SB, Cameron CL Coping through emotional approach: scale construction and validation. J Pers Soc Psychol 2000;78:1150–69. 10.1037/0022-3514.78.6.1150 10870915

[46] Brooks R EuroQol: the current state of play. Health Policy 1996;37:53–72. 10.1016/0168-8510(96)00822-6 10158943

[47] Frost RO, Marten P, Lahart C The dimensions of perfectionism. Cognitive Therapy Research 1990;14:449–68. 10.1007/BF01172967

[48] Mountford V, Corstorphine E, Tomlinson S Development of a measure to assess invalidating childhood environments in the eating disorders. Eat Behav 2007;8:48–58. 10.1016/j.eatbeh.2006.01.003 17174851

[49] Kim Y, Pilkonis PA Selecting the most informative items in the IIP scales for personality disorders: an application of item response theory. J Personal Disord 1999;13:157–74. 10.1521/pedi.1999.13.2.157 10372349

[50] Leon AC, Solomon DA, Mueller TI The Range of Impaired Functioning Tool (LIFE-RIFT): a brief measure of functional impairment. Psychological medicine: a journal of research in psychiatry and the allied sciences 1999;29:869–78. 10.1017/S0033291799008570 10473314

[51] Parker G, Roussos J, Hadzi-Pavlovic D The development of a refined measure of dysfunctional parenting and assessment of its relevance in patients with affective disorders. Psychol Med 1997;27:1193–203. 10.1017/S003329179700545X 9300523

[52] Miller IW, Norman WH, Bishop SB The Modified Scale for Suicidal Ideation: reliability and validity. J Consult Clin Psychol 1986;54:724–5. 10.1037/0022-006X.54.5.724 3771893

[53] McCrae RR, Costa PT Jr A contemplated revision of the NEO Five-Factor Inventory. Personality Individual Differences 2004;36:587–96. 10.1016/S0191-8869(03)00118-1

[54] Watson D, Clark LA, Tellegen A Development and validation of brief measures of positive and negative affect: the PANAS scales. J Pers Soc Psychol 1988;54:1063–70. 10.1037/0022-3514.54.6.1063 3397865

[55] Kroenke K, Spitzer RL, Williams JBW The PHQ-9: validity of a brief depression severity measure. J Gen Intern Med 2001;16:606–13. 10.1046/j.1525-1497.2001.016009606.x 11556941 PMC1495268

[56] Neuberg SL, Newsom JT Personal need for structure: Individual differences in the desire for simpler structure. J Pers Soc Psychol 1993;65:113–31. 10.1037/0022-3514.65.1.113

[57] Linehan MM Suicidal behaviors questionnaire. Seattle, WA: Univesity of Washington, 1981.

[58] First MB, Gibbon M, Spitzer RL Structured clinical interview for DSM-IV Axis II personality disorders, (SCID-II). Washington, D.C: American Psychiatric Press, Inc., 1997.

[59] Neff KD The development and validation of a scale to measure self-compassion. Self Identity 2003;2:223–50. 10.1080/15298860309027

[60] Sarason IG, Sarason BR, Shearin EN A Brief Measure of Social Support: practical and theoretical implications. J Soc Pers Relationships 1987;4:497–510. 10.1177/0265407587044007

[61] Schwartz SH Universals in the content and structure of values: theoretical advances and empirical tests in 20 countries. In: Zanna MP, ed. Advances in experimental social psychology, Vol 25. San Diego, CA, US: Academic Press, 1992:1–65.

[62] Whiteside SP, Lynam DR, Miller JD Validation of the UPPS impulsive behaviour scale: a four-factor model of impulsivity. European J Pers 2005;19:559–74. 10.1002/per.556

[63] Wegner DM, Zanakos S Chronic thought suppression. J Pers 1994;62:615–40. 10.1111/j.1467-6494.1994.tb00311.x 7861307

[64] Scott NW, McPherson GC, Ramsay CR The method of minimization for allocation to clinical trials: a review. Controlled Clinical Trials 2002;23:662–74. 10.1016/S0197-2456(02)00242-8 12505244

[65] Russell D, Hoare ZSJ, Whitaker R Generalized method for adaptive randomization in clinical trials. Stat Med 2011;30:922–34. 21284014 10.1002/sim.4175

[66] Emsley R, Dunn G, White I Mediation and moderation of treatment effects in randomised controlled trials of complex interventions. Stat Methods Med Res 2010;19:237–70.. 10.1177/0962280209105014 19608601

[67] Ten Have TR, Joffe MM, Lynch KG Causal mediation analyses with rank preserving models. Biometrics 2007;63:926–34. 10.1111/j.1541-0420.2007.00766.x 17825022

[68] Chen L, Smith GD, Harbord RM Alcohol intake and blood pressure: a systematic review implementing a Mendelian randomization approach. PLoS Med 2008;5:e52–00. 10.1371/journal.pmed.0050052 18318597 PMC2265305

[69] Bloom HS Learning more from social experiments: evolving analytic approaches. Russell Sage Foundation, 2005.

[70] Miller SD, Duncan BL, Brown J Using formal client feedback to improve retention and outcome: making ongoing, real-time assessment feasible. J Brief Therapy 2006;5:5–22.

[71] Stock JH, Wright JH, Yogo M A survey of weak instruments and weak identification in generalized method of moments. J Business Econ Stat 2002;20.

[72] Thompson ER Development and validation of an internationally reliable Short-Form of the Positive and Negative Affect Schedule (PANAS). J Cross-Cultural Psychol 2007;38:227–42. 10.1177/0022022106297301

[73] Harley R, Sprich S, Safren S Adaptation of dialectical behavior therapy skills training group for treatment-resistant depression. J Nerv Ment Dis 2008;196:136–43. 10.1097/NMD.0b013e318162aa3f 18277222

[74] Wampold BE, Minami T, Baskin TW A meta-(re) analysis of the effects of cognitive therapy versus ‘other therapies’ for depression. J Affect Disord 2002;68:159–65. 10.1016/S0165-0327(00)00287-1 12063144

[75] Baron RM, Kenny DA The moderator–mediator variable distinction in social psychological research: conceptual, strategic, and statistical considerations. J Pers Soc Psychol 1986;51:1173–82. 10.1037/0022-3514.51.6.1173 3806354

[76] NICE. (National institute for health and clinical excellence). Depression: the treatment and management of depression in adults (update). London: National Institute for Health and Clinical Excellence, 2009.

[77] Fenwick E, Byford S A guide to cost-effectiveness acceptability curves. Br J Psychiatry 2005;187:106–8. 10.1192/bjp.187.2.106 16055820

